# Fatty Acids Derived from Royal Jelly Are Modulators of Estrogen Receptor Functions

**DOI:** 10.1371/journal.pone.0015594

**Published:** 2010-12-22

**Authors:** Paraskevi Moutsatsou, Zoi Papoutsi, Eva Kassi, Nina Heldring, Chunyan Zhao, Anna Tsiapara, Eleni Melliou, George P. Chrousos, Ioanna Chinou, Andrey Karshikoff, Lennart Nilsson, Karin Dahlman-Wright

**Affiliations:** 1 Department of Biological Chemistry, Medical School, University of Athens, Athens, Greece; 2 Department of Biosciences and Medical Nutrition, Karolinska Institutet, NOVUM, Huddinge, Sweden; 3 Division of Pharmacognosy and Natural Products, Faculty of Pharmacy, University of Athens, Athens, Greece; 4 First Department of Pediatrics, Medical School, University of Athens, “Aghia Sophia” Children's Hospital, Athens, Greece; New Mexico State University, United States of America

## Abstract

Royal jelly (RJ) excreted by honeybees and used as a nutritional and medicinal agent has estrogen-like effects, yet the compounds mediating these effects remain unidentified. The possible effects of three RJ fatty acids (FAs) (10-hydroxy-2-decenoic-10H2DA, 3,10-dihydroxydecanoic-3,10DDA, sebacic acid-SA) on estrogen signaling was investigated in various cellular systems. In MCF-7 cells, FAs, in absence of estradiol (E_2_), modulated the estrogen receptor (ER) recruitment to the pS2 promoter and pS2 mRNA levels via only ERβ but not ERα, while in presence of E_2_ FAs modulated both ERβ and ERα. Moreover, in presence of FAs, the E_2_-induced recruitment of the EAB1 co-activator peptide to ERα is masked and the E_2_-induced estrogen response element (ERE)-mediated transactivation is inhibited. In HeLa cells, in absence of E_2_, FAs inhibited the ERE-mediated transactivation by ERβ but not ERα, while in presence of E_2_, FAs inhibited ERE-activity by both ERβ and ERα. Molecular modeling revealed favorable binding of FAs to ERα at the co-activator-binding site, while binding assays showed that FAs did not bind to the ligand-binding pocket of ERα or ERβ. In KS483 osteoblasts, FAs, like E_2_, induced mineralization via an ER-dependent way. Our data propose a possible molecular mechanism for the estrogenic activities of RJ's components which, although structurally entirely different from E_2_, mediate estrogen signaling, at least in part, by modulating the recruitment of ERα, ERβ and co-activators to target genes.

## Introduction

**Figure 1:**
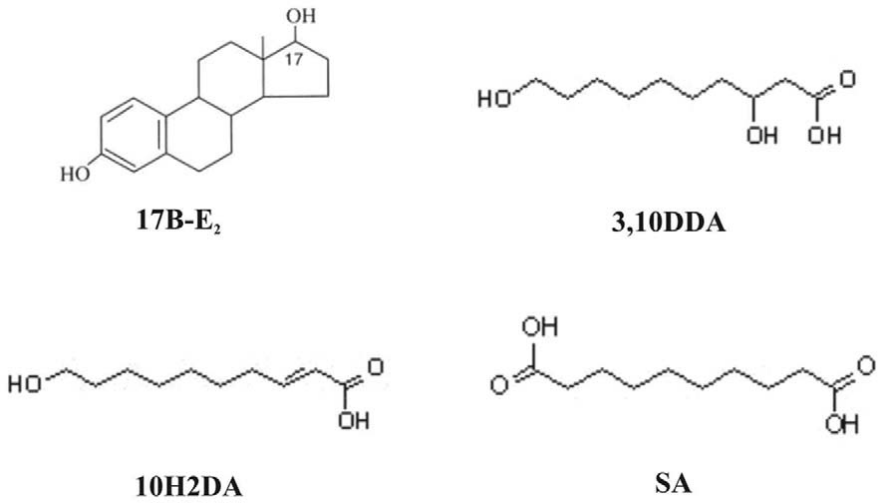
Structures of 17β-estradiol (17β-E_2_), 10-hydroxy-2-decenoic acid (10H2DA), 3,10-dihydroxydecanoic acid (3,10DDA) and sebacic acid (SA).

Royal jelly (RJ), a yellowish material excreted by the mandibular and hypopharyngeal glands of worker bees of the genus *Apis mellifera*, is a food essential for the longevity of the queen bee. RJ exerts estrogen effects in vitro and in vivo, similar to those evoked by 17β-estradiol (E_2_) [1], [2], [3]. However, the mediators of RJ's estrogenic effects remain unknown. While RJ contains a considerable amount of proteins, free amino acids, sugars, vitamins and sterols, the medium chain fatty acids (FAs) 10-hydroxy-2-decenoic (10H2DA), 3,10-dihydroxydecanoic (3,10 DDA) and sebacic (SA) acids (Fig. 1) are major and unique RJ components [4], [5], [6].

Estrogens play pivotal roles in regulating the function of many tissues and organs and estrogen signaling has been associated with a number of diseases, including breast and uterine cancers, disorders of lipid metabolism, cardiovascular diseases, autoimmune inflammatory diseases, osteoporosis, menstrual abnormalities and infertility [7]. Estrogens exert their effects via intracellular receptors, estrogen receptors alpha (ERα) and beta (ERβ) [8], [9], [10]. In the presence of ligands, both ERα and ERβ are activated and as dimers interact with specific DNA sequences. Activated ERs interact with other nuclear proteins, such as steroid receptor co-regulators, altering the transcription rates of responsive genes. The activated ERα and ERβ can also bind to other transcription factors, such as activator protein 1 (AP-1) and nuclear factor kappa B (NF-kB), affecting their binding to their cognate DNA sequences and their transcriptional effects [11]. More recently, the G protein-coupled receptor, GPR30/GPER, has been shown to mediate rapid estrogen effects as well as to regulate transcriptional activation. Possible synergism and antagonism with classical estrogen receptors has been suggested [12].

In the present study, we investigated the possible estrogenic/antiestrogenic effects of the RJ-derived fatty acids, 10H2DA, 3,10DDA and SA, in various cellular systems in vitro. We examined the ability of FAs, at physiologically achievable levels, to modulate 1) the recruitment of ERα and ERβ to the E2 responsive region of the pS2 promoter in the MCF-7 cell line, 2) the regulation of pS2 mRNA levels in the MCF-7 cell line, 3) the activity of ERα and ERβ on an ERE-driven Luc-reporter gene in MCF-7 and HeLa cells and 4) the E_2_-induced recruitment of the EAB1 co-activator peptide to ERα. Furthermore, we examined the potential of FAs to induce mineralization in KS483 osteoblasts, which is an ER regulated process in bone remodeling. Finally, we assessed the capacity of FAs to bind to ERs and we also modeled the interaction of FAs with ERα to reveal potentional sites of interaction.

## Materials and Methods

### 1. Isolation and identification of fatty acids

The 10-hydroxydec-2-enoic (10H2DA), 3,10-dihydroxydecanoic (3,10DDA) and sebacic (SA) fatty acids were isolated from RJ by chromatographic separation (Liquid Chromatography, LC and Medium Pressure Liquid Chromatography, MPLC) and identified by means of spectroscopic data analysis, mainly via the concerted application of 1D and 2D Nuclear Magnetic Resonance (NMR) techniques (Heteronuclear Multiple Quantum Coherence, HMQC and Heteronuclear Multiple Bond Coherence, HMBC) and mass spectrometry, as described previously [6].

### 2. Cell cultures

A cervical adenocarcinoma ER negative cell line (HeLa, ATCC Cell Bank), an endometrial ER positive cancer cell line (Ishikawa ECACC Cell Bank, No 99040201), an ERα positive breast carcinoma cell line (MCF-7, ATCC Cell Bank) and a human hepatoma ER negative cell line (Huh7, ATCC Cell Bank) were used. For chromatin immunoprecipitation (ChIP) experiments, a stable cell line, MCF-7 tet-off Flag-ERβ that expresses an inducible version of ERβ fused to a Flag-tag, was used. This cell line expresses endogenous ERα. The KS483 bone cell line is a non-transformed stable subclone of a parental mouse cell line KS4 that has the ability to form mineralized nodules in vitro. All cell lines were maintained as previously described [13], [14], [15].

### 3. Chromatin immunoprecipitation assay (ChIP)

Cells were seeded in 150-mm dishes and grown in the presence (ERα+/ERβ−) or in the absence of tetracycline (ERα+/ERβ+) for 4 days in phenol red (PR) free DMEM supplemented with 10% dextran-coated charcoal (DCC)-treated fetal bovine serum (FBS). Cells were treated with 10^−8^ M E_2_ or 10^−6^ M FAs for 45 min. Co-incubation was performed with 10^−8^ M E_2_ and 10^−6^ M FAs. ChIP was performed as previously described [14], [16]. The anti-ERβ rabbit polyclonal antibody LBD [17] was used to perform ChIP for ERβ and the rabbit polyclonal anti-ERα antibody HC-20 was used for ERα ChIP. Normal rabbit IgG was used for determination of non-specific binding. The final ChIP DNA was amplified by real-time PCR with SYBR green master mix RT-PCR reagent, using primers that amplify the ER binding region from the pS2 promoter. 18s was used as negative control. The primer pairs are listed in Table 1.

**Table 1:** Primer pairs for amplification of ChIP enriched regions of pS2 promoter and 18s and mRNA levels of ERα, pS2 and acidic ribosomal phosphoprotein PO (36B4).

		Forward	Reverse
**ChIP**	**18S**	GCTTAATTTGACTCAACACGGGA	AGCTATCAATCTGTCAATCCTGTC
	**pS2**	CCT CCC GCC AGG GTA AAT AC	CCG GCC ATC TCT CAC TAT GAA
**mRNA**	**ERα**	GAA TCT GCC AAG GAG ACT CGC	ACT GGT TGG TGG CTG GAC AC
	**pS2**	CATCGACGTCCCTCCAGAAGAG	CTCTGGGACTAATCACCGTGCTG
	**36B4**	GTG TTC GAC AAT GGC AGC AT	GAC ACC CTC CAG GAA GCG A

### 4. Determination of mRNA and protein levels

Cells were seeded in 6-well plates and grown in the presence (ERα+/ERβ−) or in the absence of tetracycline (ERα+/ERβ+) for 4 days in PR free DMEM 10% DCC-FBS. Cells were treated with 10^−8^ M E_2_ or 10^−10^–10^−5^ M FAs for 24 hrs. Co-incubation was performed with 10^−8^ M E_2_ and 10^−9^, 10^−7^ or 10^−6^ M FAs. Total RNA were purified using the RNeasy Mini Kit. Two µg of total RNA was reverse transcribed into cDNA using TaqMan Reverse Transcription Reagents with random hexamer primers. Real time PCR assays were conducted using SYBR green master mix RT-PCR reagent. Acidic ribosomal phosphoprotein PO (36B4) was used as an internal control gene [18]. The sequences of the primers are listed in Table 1. For detecting ERα protein levels, cells were incubated as mention above. Western blot analysis was carried out as previously described [19] using the following antibodies: anti-ERα (HC-20, Santa Cruz Biotechnology) and anti-β actin (A2228, Sigma).

### 5. Transfection studies in HeLa cells and MCF-7 cells

Before each transfection experiment cells were maintained for 2 days in PR free DMEM containing 10% DCC-FBS. For transfection assays, cells were plated in 6-well or 24-well plates in PR free DMEM with 10% DCC-treated FBS and transfected using reagents and plasmids as stated in Table 2, according to the manufacturer's instructions and as previously described [13]. MCF-7 cells transfected with EREs were incubated with E_2_ (10^−8^ M) or FAs (10H2DA, 3,10DDA, SA) in a concentration range of 10^−10^–10^−5^ M. Co-incubation of FAs with E_2_ (10^−8^ M) was also carried out. MCF-7 cells transfected with Glucocorticoid Respone Element (GRE) were incubated with dexamethasone (DEX) (10^−6^ M), RU486(10^−6^ M) or FAs (10H2DA, 3,10DDA, SA) in a concentration range of 10^−10^–10^−5^ M. Co-incubation of FAs (10^−7^ M) with DEX (10^−6^ M) was also performed. HeLa cells transfected with ERα or ERβ were incubated with E_2_ (10^−9^ M) or ICI182780 (10^−8^) or 4OH-tamoxifen (4OH-TMX) (10^−8^ M) or FAs (10H2DA, 3,10DDA, SA) in a concentration range of 10^−10^–10^−5^ M. Co-incubation of E_2_ (10^−9^ M) with ICI182780 (10^−8^ M) or FAs (10^−7^–10^−6^ M) was also conducted. Cells were harvested 24 hrs later and cell extracts were assayed for luciferase, β-galactosidase and renilla luciferases, as stated in Table 2.

**Table 2:** Transfection conditions used in HeLa and MCF7 cells. Plasmids and reagents are listed accordingly.

	Plasmids	DNA quantity/well	Reagents
**MCF7-ERE**	ERE (2xERE-TATA-Luc)	0.2 µg	Lippofectamine (Invitrogen)
	pRL-TK (Renilla-Promega)	0.01 µg	
**MCF7-GRE**	GRE (MMTV-Luc)	0.2 µg	Effectene Tranfection Reagent (Qiagen)
	β-gal (pCMVβ)	0.2 µg	
**HeLa-ERα**	ERα (HO-hERα)	0.5 µg	Polyfect Transfection Reagent (Qiagen)
	ERE (3xERE-TATA-Luc)	0.5 µg	
	β-gal (pCMVβ-Clontech)	0.5 µg	
**HeLa-ERβ**	ERβ (pSG5-hERα)	0.5 µg	Polyfect Transfection Reagent (Qiagen)
	ERE (3xERE-TATA-Luc)	0.5 µg	
	β-gal (pCMVβ-Clontech)	0.5 µg	

### 6. Mammalian two-hybrid assay

The day before the transfection, Huh7 cells were seeded into 24-well plates in PR free medium 10% DCC-FBS and 2 mM L-glutamine. Cells were transfected with Genejuice as instructed by the manufacturer. After transfection, cells were treated with E_2_ (1 µM), 4OH-TMX (500 nM), FAs (5 µM) or FAs in combination with E_2_ for 16 h. C. Luciferase and β-galactosidase activity was assayed as earlier described [15].

### 7. Mineralization assay in KS483

For the assays, cells were seeded in 12-well plates in a-MEM 10% DCC-FBS. Three days after plating, cells reached confluence and were subsequently induced to differentiate by the addition to the culture medium of 50 µg/ml ascorbic acid in the absence or presence of FAs in a concentration range 10^−10^–10^−7^ M. E_2_ (10^−9^–10^−6^ M) was used as positive control. Co-incubation with ICI182780 (10^−7^ M) was also performed. B-glycerophosphate was added after day 10. The medium with the reagents was refreshed every 3–4 days for 24 days in total. After 24 days, cells were rinsed with PBS. The number of mineralized bone nodules was identified with Alizarin Red-S. For Alizarin Red- S (sodium alizarin sulphonate) staining, 2% Alizarin Red- S (Sigma) was prepared in distilled water and the pH was adjusted to 5.5. Cultures were fixed with 5% formalin (10 min), washed, and stained with Alizarin Red- S for 5 min. After removal of unincorporated excess dye with distilled water, the mineralized nodules were labeled as red spots. Mineralized nodules were counted by light microscopy at a 10-fold magnification as described previously [13], [20].

### 8. MTT cell viability assay

Ishikawa cells and MCF-7 were cultured and the effect of FAs (1.6×10^−7^–4×10^−4^ M) on cell viability was estimated by a modification of the MTT assay, as previously described [13]. This assay measures the fraction of active mitochondria of living cells. Thus, since results depend both on the mitochondria activity per cell and on the number of cells present, MTT assay estimates cell proliferation and survival [21].

### 9. Ligand binding assay

The ligand binding domain of the human ERα (hERα-LBD) and human ERβ (hERβ-LBD) were produced individually in *Escherichia coli* in 2xLB medium supplemented with 50 µM biotin. The cells were harvested by centrifugation and the cell pellet stored frozen at −20°C. The pellets were suspended in Tris buffer and the cell walls were disrupted in a Microfluidizer M-110L. The supernatants with receptor were stored at −70°C. The expression of recombinant ERα and ERβ, respectively, in the extracts was confirmed using the ERα selective agonist PPT (propylpyrazol triol) and the ERβ selective agonist DPN (2,3,-bis(4-hydroxyphenyl) propionitrile) [22], [23]. Receptor extracts were thawed on ice from −70°C and mixed with streptavidin coated SPA beads in pH8 buffer (1 mM EDTA, 18 mMK_2_HPO_4_, 2 mM KH_2_PO_4_, 20 mM Na_2_MoO_4_, 1 mM TCEP). The compounds were diluted in DMSO to 12 concentrations and 18 µl of each dilution was added in duplicates to a Corning 3706 plate. The final assay concentration of tracer was 1.2+/−0.08 nM and the compound concentrations ranged from 37 pM to 157 µM in a total volume of 88 µl. The plates were incubated on a shaker overnight at room temperature, centrifuged (2000 rpm, 5 min) and measured with top and bottom detectors on 12 detector Trilux Microbeta. A four parameter logistic fit (4PL) was used to analyze the data with XLfit software from IDBS in Microsoft Excel.

### 10. Modeling of fatty acid interactions with ERα

Three-dimensional models of the FAs (10H2DA, 3,10 DDA, and SA), as well as of the co-factor peptide EAB1, were built using PyMol. The FAs were docked to the ligand pocket and to the co-activator binding site and then the complexes were minimized using 100 steps of Steepest Descent followed by 500 steps of Adopted Basis Newton-Raphson minimization in CHARMM [24]. The parameters for the FAs were compiled using the CHARMM force field for proteins [25], lipids [26], [27] and the CHARMM general force field [28]. The X-ray structure of the ERα receptor with PDB entry code 1GWR [29], [30] was used in the calculations. Missing atoms were built and E_2_ was parameterized as previously described [31]. The binding of the organic molecules to the receptor was evaluated on the basis of the interaction energy (Coulomb and van der Waals interactions) between receptor and ligand or cofactor peptide.

## Results

The RJ's FAs may modulate estrogen signaling by various mechanisms, involving binding to the ligand binding pocket of the receptor, influencing the abundance/distribution of ER subtypes and their recruitment to E_2_ responsive genes, modulating co-activators and/or co-repressors, physically blocking co-activator and co-repressor recruitment, or alternatively by inducing proteins which may disrupt ER dimerization. Estrogenic effects of RJ FAs could also involve GPR30-mediated signaling [12]. We investigated the RJ FAs with regard to effects on a panel of in vitro bioassays that detect estrogenicity/antiestrogenicity of a test substance [21], [32].

We examined the estrogenic/antiestrogenic activity of 10H2DA, 3,10DDA and SA, which were isolated and identified previously [6], in several estrogen-responsive biological systems (Fig. 1). E_2_ was used as positive control for agonist activity, whereas ICI182780, a well-known complete estrogen antagonist, served as control for antagonist action. 4OH-TMX served as control for partial estrogen agonism/antagonism activity.

### FAs induce ERβ recruitment to the pS2 promoter

**Figure 2:**
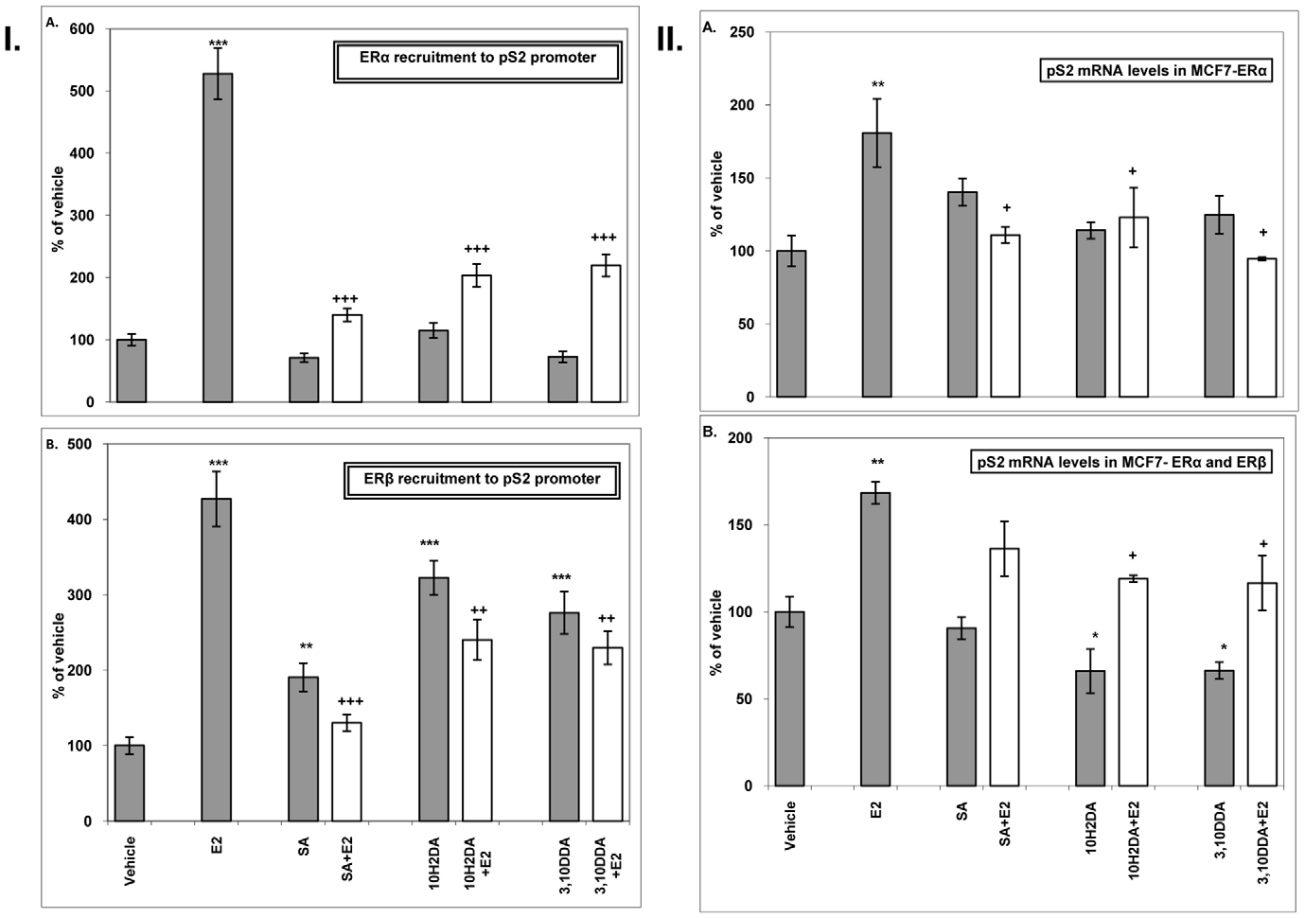
Effects of FAs on ERα (A) and ERβ (B) recruitment to the pS2 promoter (I). Effects of FAs on pS2 mRNA levels in the presence of ERα (A) or ERα and ERβ (B) together (II). MCF-7 tet-off Flag-ERβ cells were treated as mentioned in Materials and Methods. Results are expressed as fold binding compared to vehicle and normalized to recruitment of ERs to the 18S gene (I) and 36B4 (II). Treatment with 10^−5^ M of FAs gave similar results (data not shown). Mean values ± SD are shown from three independent experiments. *Significantly different from vehicle (*p<0.05, **p<0.01, ***p<0.001), +significantly different from E_2_ (10^−8^ M) ((+p<0.05, ++p<0.01, +++p<0.001).

Figure 2.I. shows the effects of FAs on ERα (A) and ERβ (B) recruitment to the pS2 gene promoter. FAs did not induce ERα recruitment to the pS2 promoter (Fig. 2.I.A). As expected, E_2_ (10^−8^ M) enhanced recruitment of ERα to the pS2 promoter (Fig. 2.I.A). However, co-incubation of either FA (10^−6^ M) with E_2_ (10^−8^ M) inhibited E_2_-dependent recruitment of ERα to the pS2 promoter. Fig. 2.I.B shows that all FAs and E_2_ (10^−8^ M) increase recruitment of ERβ to the pS2 promoter. However, upon co-incubation of either FAs at 10^−6^ M with E_2_ (10^−8^ M), decreased recruitment compared to that observed for E_2_ alone is observed for ERβ to the pS2 promoter (p<0.01–0.001).

### FAs modulate pS2 mRNA levels

In the presence of ERα, FAs at all concentrations tested did not change pS2 mRNA levels, while pS2 mRNA levels were increased after E_2_ treatment (Fig. 2.II.A). However, when co-incubated (10^−6^ M) with E_2_, FAs decreased E_2_-mediated induction of pS2 mRNA consistent with the results of ChIP assay. When ERβ was co-expressed with endogenous ERα, 10H2DA and 3,10DDA significantly decreased pS2 mRNA levels at concentrations of 10^−6^ M (Fig. 2.II.B). In this system, 10H2DA and 3,10DDA also abolished the induction of pS2 mRNA by E_2_. In MCF-7 cells, with or without ERβ expression, FAs alone, at all concentrations tested, do not affect ERα mRNA or nuclear ERα protein levels (Fig. S1).

### FAs reduce ERE-mediated transcriptional activity in MCF7 cells

**Figure 3:**
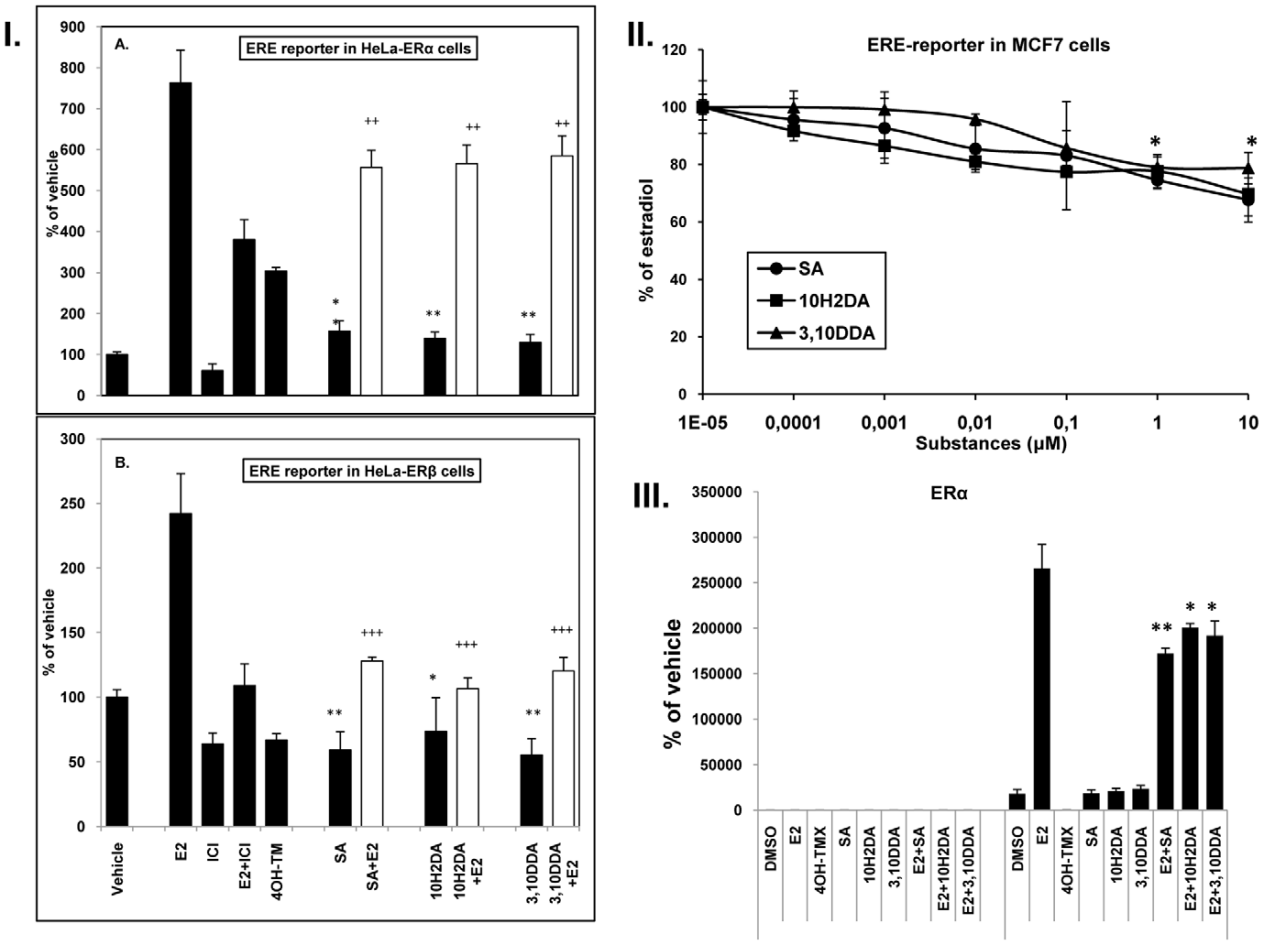
Effects of FAs on ERE-mediated transactivation in HeLa cells transfected with ERα (A) or ERβ (B) (I). Effects of FAs, on ERE-mediated transactivation in MCF-7cells (II). Cells were transfected under conditions as shown in Table 2 and treated as mentioned in Materials and Methods. Results represent the mean ± SD of three independent experiments. *Significantly different from vehicle (*p<0.05, **p<0.01, ***p<0.001), +significantly different from E_2_ (10^−8^ M) (+p<0.05, ++p<0.01, +++p<0.001). Analysis of ER-co-activator peptide (EAB1) interaction with mammalian two-hybrid assay (III). Huh7 cells were transiently transfected and treated as mentioned in Materials and Methods. Luciferase activity was normalized to β-galactosidase activity. Mean values ± SE are shown from the results of four independent experiments (* p<0.05 or p<0.01 significantly different from E_2_ (10^−6^ M).

The addition of 10H2DA, 3,10DDA or SA (10^−10^–10^−5^ M), in the presence of E_2_ (10^−8^ M), inhibited the E_2_-mediated induction of an ERE-driven luciferase reporter gene in MCF-7 cells in a dose-dependent manner (Fig. 3.II). When incubated in the absence of E_2,_ all FAs increased slightly, but not significantly, the basal ERE-driven luciferase activity, in the concentration range of 10^−6^–10^−5^ M (Fig. S2). In MCF-7 cells transfected with GRE-driven luciferase reporter, the addition of 10H2DA, 3,10DDA or SA (10^−10^–10^−5^ M) did not alter the GRE-mediated transcriptional activity, when assayed alone or in the presence of DEX (10^−6^ M) (Fig. S3).

### FAs modulate ERα- and ERβ-mediated reporter gene activity in HeLa cells

The ability of E_2_, ICI182780, 4OH-TMX and FAs to modulate ERE-driven luciferase activity in HeLa cells transfected with either ERα (A) or ERβ (B) is shown in Figure 3.I. The presence of E_2_ (10^−9^ M) increased the ERα- and ERβ-mediated luciferase activity, while co-incubation with ICI182780, as expected, diminished the E_2_-enhancing effect in both systems. ICI182780 (10^−8^ M), when added alone, diminished the basal luciferase activity mediated by ERα and ERβ. In agreement with previous reports, 4ΟΗ-TMX was a weak agonist of ERα and a potent antagonist of ERβ in this system [33]. All FAs enhanced the ERα-mediated activity, when incubated alone at various concentrations (10^−10^–10^−5^ M) (Fig. S4). Moreover, FAs attenuated the effects of E_2_ under co-incubation conditions (Fig. 3.I.A). All FAs diminished ERβ-mediated activity when incubated alone at various concentrations (10^−10^–10^−5^ M) (Fig. S4). These FAs also attenuated the effects of E_2_ under co-incubation conditions (Fig. 3.I.B). Figure 3.I shows the data for the effects of FAs on ERE-luciferase activity at a FAs concentration of 10^−6^ M and co-incubation with 10^−9^ M E_2_ (full data in Fig. S4).

### FAs alter E_2_- induced co-activator recruitment to ERα

The molecular basis for ER agonism is dependent on formation of a hydrophobic surface within the LBD, which represents the docking surface for α-helical leucine-rich peptide motifs in co-activators [29]. A mammalian two-hybrid assay was used to monitor induction of an agonist conformation in the receptor, which allows recruitment of a peptide containing an α-helical leucine-rich motif (LxxLL) upon ligand binding [15]. The LxxLL-containing peptide EAB1 is strongly associated with the receptor when E_2_ is added, indicating a structural change where the receptor adopts an agonist conformation. The fatty acids, while alone, do not induce a detectable conformational change in ERα. However, when the fatty acids are co-incubated with E_2_, recruitment of the LxxLL peptide is diminished (Fig. 3.III).

### FAs induce mineralization in osteoblasts

**Figure 4:**
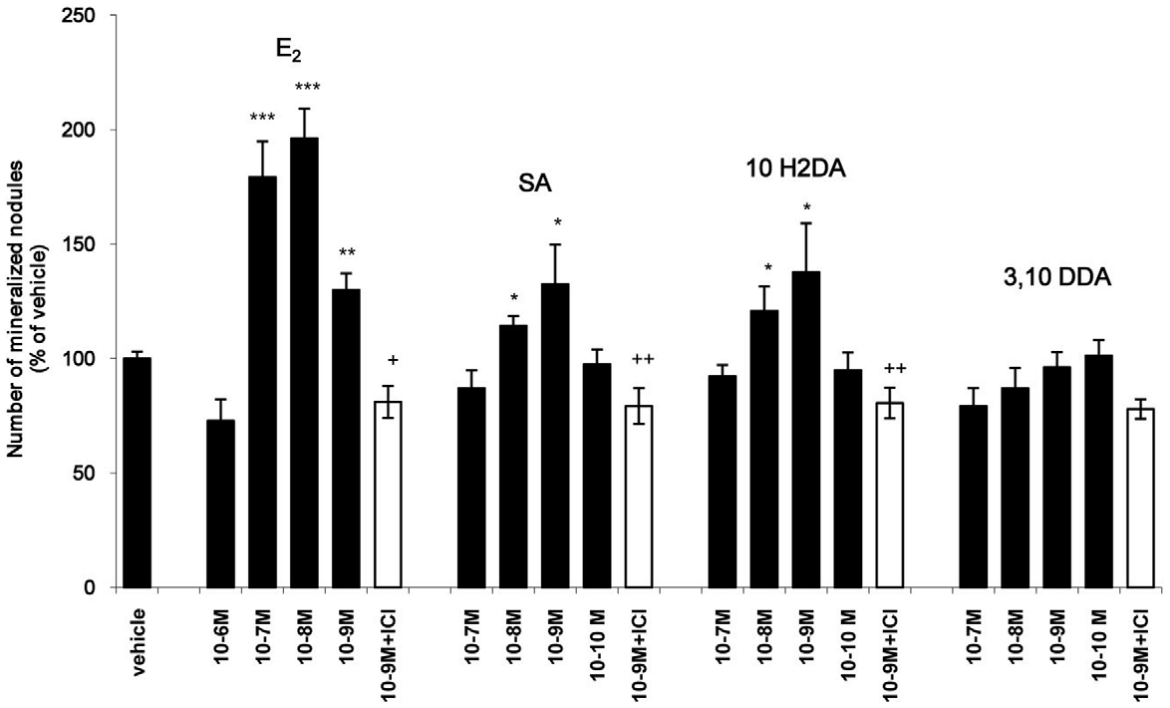
Effect of E_2_ and FAs on mineralization of KS483 cells. Cells were treated as mentioned in Materials and Methods. Results are expressed as percentage of vehicle. Mean values ± SD are shown from the results of three independent experiments. (* p<0.05 or **p<0.01 or ***p<0.001 significantly different from vehicle, + p<0.05 or ++p<0.01 significantly different from E_2_ (10^−9^ M) or FAs (10^−9^ M).

As shown in Fig. 4, the presence of E_2_ (10^−9^–10^−8^ M) induced mineralization in osteoblasts, as expected [20]. Similarly, 10H2DA and SA at 10^−9^–10^−8^ M exhibited an agonistic effect by inducing nodule formation, an effect which was diminished in the presence of ICI182780, thereby suggesting an ER-mediated action.

### FAs do not bind to ERα or ERβ

To examine a possible binding of FAs to the ligand pocket of the receptor, we used a competition binding assay. Using ERα (PPT) and ERβ (DPN) selective agonists, we confirmed the expression and specificity of the receptors in the cell extracts used in this assay. PPT exhibited 1000-fold higher relative binding affinity in ERα- than in ERβ-expressing cell extracts (10^−9^ M and 10^−6^ M respectively), while DPN had 200-fold higher relative binding affinity in ERβ-expressing cell extracts compared to ERα-expressing cell extracts (10^−8^ M). E_2_ had equal Relative Binding Affinity (RBA) in both cell extracts (10^−9^). The assays revealed that SA and 3,10DDA did not bind to ERα or ERβ at all concentrations tested (data not shown). However, 10H2DA exhibited binding to both receptors, but only at extreme concentrations (10^−4^ M).

### Modeling of FA interactions with ERα

**Figure 5:**
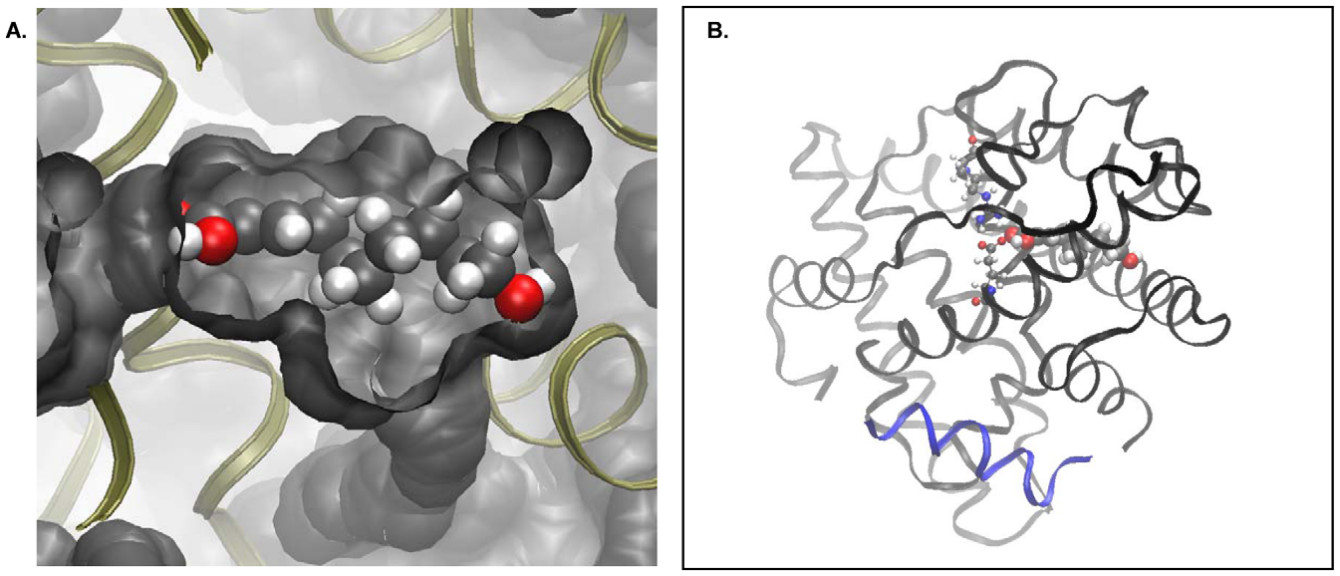
Modeling of interactions of fatty acids with ERα. A. The fatty acid 10H2DA in the ligand pocket of ERα. The protein molecule is represented by its contact surface, whereas the fatty acid is represented by spheres (oxygen atoms in red, carbon atoms in grey). B. The pair Glu353-Arg394 (residue numeration follows that of PDB entry 1GWR) and the carboxyl group of 10H2DA (van der Waals spheres) in the ligand pocket of the estrogen receptor. The orientation of the protein molecule is identical to that in A. The co-activator EAB1 is represented by ribbon in blue.

The FAs were docked in the ERα ligand binding pocket, with the EAB1 peptide present at the co-activator binding site, and interaction energies between FAs and ERα were obtained in the range of −44 to −63 kcal/mol. For comparison, the interaction energy between the receptor molecule and E_2_ obtained by the same computational procedure is -70 kcal/mol (Fig. **5**). We also docked SA at the co-activator binding site, replacing EAB1. In this case also, the interaction energy between the two molecules was favorable (about -140 kcal/mol). However, when SA was docked at other locations on the protein surface, distant from the co-activator binding site, the interaction energy turned out to be similar or even more favorable (data not shown).

## Discussion

In this study, we determined the possible estrogenic/antiestrogenic properties of 10H2DA, 3,10DDA and SA, isolated from RJ and identified by spectroscopic methods [6]. In choosing the concentrations we considered 1) the commonly used RJ dietary supplementation (1–3 g daily), 2) the concentration of 10H2DA and the concentration of sebacic acid in RJ (3–6% and 0.5% respectively) [34], [35], 3) the concentration of 10H2DA, sebacic acid and 3,10 DDA as well as 10HDA acid in marketed RJ samples in Greece (40–50%, 5%, 4% and 20% respectively), 4) the human blood volume and bioavailability. Based on the above information, we decided to examine the biological effects of FAs in a concentration range of 10^−10^ M–10^−5^ M, which are physiologically achievable concentrations.

Using a ChIP assay in MCF-7 breast cancer cells, which are stably transfected with an inducible version of ERβ and express endogenous ERα, we examined the ligand-dependent recruitment of ERα and ERβ to chromatin. None of the tested FAs could modulate ERα recruitment to the pS2 promoter, whilst they increased ERβ recruitment to this promoter. All FAs inhibited the effect of E_2_ on ERα and ERβ recruitment. Consistent with the effects on receptor recruitment to DNA, experiments revealed that in the presence of ERβ, FAs could decrease pS2 mRNA levels, when added alone, and that they decreased E_2_'s effect in the presence and absence of ERβ. However, since in this cell system endogenous ERα is always present, effects on pS2 expression cannot easily be determined for ERβ alone. We further assessed the effects of FAs on ERα alone and ERβ alone in HeLa cells. This cell line, in contrast to MCF-7 cells, lacks endogenous ER. In HeLa cells, we demonstrated that all FAs, when assayed alone, were weak enhancers of ERα-mediated activity, while they antagonized ERβ-mediated effects. In the presence of E_2_ they antagonized the E_2_-mediated effects via ERα and ERβ. The well characterized selective estrogen receptor modulator (SERM) 4OH-TMX also exhibited agonistic effects on ERα-mediated activity, while it was a complete antagonist of ERβ-mediated action. This is in agreement with a previous study reporting that 4OH-TMX induced ERE-mediated reporter gene activity in a stably transformed ERα expressing cell line, but exhibited pure antagonism in the corresponding ERβ expressing system [33].

Recruitment of co-factors is an essential component of ER signaling. The best defined structure-function of a co-regulator interaction is with co-activators that interact through a conserved LxxLL motif, termed an NR box. Interestingly, in MCF-7 cells we show that the recruitment of the EAB1 co-activator peptide upon E_2_ binding is reduced when FAs are present. This suggests that FAs are preventing proper ER activity, possibly by inducing a conformational response at the co-activator binding site, leading to masking of the co-activator site.

In the ERE-driven luciferase reporter gene assay in MCF-7 cells, all 3 FAs inhibited the E_2_-mediated increase in luciferase activity, suggesting an ER-mediated effect and a common signal transduction pathway for E_2_ and FAs at the level of ERE-containing promoters. Additionally, all 3 FAs showed a trend towards increasing the ERE-driven luciferase activity when tested alone. This is consistent with results from Suzuki et al. showing that 10H2DA increased the ERE-driven luciferase activity in MCF-7 cells at the same concentration range. However, co-incubation of FAs with E2 was not investigated in their study [36]. In previous reports [2] fresh RJ displays agonistic activity in the ERE-driven luciferase reporter gene assay in MCF-7 cells similar to that observed for E_2_ whereas the isolated FAs in our study show little agonist activity and possess antagonistic activity. RJ contains multiple FA components [6] and data indicate that 10H2DA, sebacic acid and 3,10 DDA (investigated in this study) may not be the only FA determinants that predict estrogen/antiestrogen activity in RJ [36]. Additionally, RJ may exhibit biological effects determined by synergistic and/or antagonistic interactions between its constituents thus showing different biological effects than the biological activity of its isolated components.

The specificity of FAs with regard to steroid receptor activation was explored by assaying the effects of FAs in MCF-7 cells on GRE-mediated transactivation. The FAs did not alter the basal nor the Dex-induced GRE-mediated transcriptional activity, indicating that the inhibition by FAs has specificity with respect to modulation of NR-mediated functions. In line with our findings, Thurmond et al. [37] proposed that medium chain FAs (hexanoate) at high concentrations (mM range) interacted with ERs to inhibit ligand stimulated transcription, while there was no effect on GR-mediated activity. Previous reports have shown that short chain FAs (valproic acid or butyrate and methoxyacetic acid) may act as deacetylase inhibitors at high concentrations (mM range) resulting in the induction of transcriptional silencing of ERα expression, which would imply that they are antiestrogenic in MCF-7 cells [38], [39], [40], [41]. The antiestrogen effects of the above short chain FAs are considered an effect that may be due to their inherent HDAC inhibitory activities, since they have all been shown to reduce endogenous ERα expression and have been characterized as HDAC inhibitors. Interestingly, a recent report showed that methoxyacetic acid (MAA at mM concentrations) modulates ERα and ERβ-mediated signaling, lowers endogenous ERα expression and antagonizes E_2_-stimulated expression of ERα target genes, yet it does not compete with E_2_ for binding to ERα [41], [42]. However, in our study, FAs (at µΜ concentrations) did not affect ERα mRNA or protein levels.

We have explored possible mechanism(s) for the effects of FAs on ER signaling by molecular modeling. As mentioned above, it is possible that the FAs compete with the LXXLL- containing co-activator for the activation function domain 2 (AF2) binding site of the receptor. Of note, docking experiments showed significant favorable interaction energy between the FAs and ERs. However, similar interaction energies were also observed for other locations on the protein's surface, distant from the co-activator binding site. Among the locations showing substantially more favorable intermolecular interactions (−211 kcal/mol) is a region including the loop around Tyr459. This loop is part of the subunit interface in the dimeric ER. Hence, binding of FAs may interfere with the dimerization of ERs and in this way influence co-activator binding (Fig.**5**).

FAs may bind to the ligand pocket, thus competing with E_2_. The computational fitting showed very good compatibility of the ligand pocket for all three FAs (Fig.**5**). Although the calculated interaction energies between ligands and receptor are only indirectly related to binding affinities, they do indicate that, similarly to E_2_, the three FAs interact favorably with the ER when they are in the ligand pocket. However, our competition binding study did not show any binding of SA and 3,10DDA and binding only at extreme concentrations (10^−4^ M) of 10H2DA, indicating that an interaction with ERs is not mediated via the ligand binding pocket. In agreement, Suzuki et al (2007) showed that 10H2DA had little effect (about 20% inhibition) upon the ability of E_2_ to bind to ERα and 50% inhibition of E_2_ to bind to ERβ at a concentration of approximately 100 µΜ [36].

In line with our findings, a recent study on 3,3′-diindolylmethane, a selective activator of ERβ that does not bind to ERβ, proposes a possible mechanism of activation through recruitment of co-activators (i.e. SRC-2) [43]. Moreover, it has been shown that the methoxyacetic study which modulates ERα signaling yet does not bind to ERα [41]. Of note, recent findings indicate that ligands, without binding affinity to ERα, activate GPR30 signaling and may act synergistically or may antagonize ERα-mediated gene expression [12]. Future studies should address the potential of FAs to activate GPR30 signaling or phosphorylation pathways in cooperation with ERs.

On the basis of the findings by Narita et al. [3] demonstrating that RJ stimulates bone formation, we used the osteoblastic cell line KS483 followed by the Alizarin Red-S staining as a model system to study the effect of FAs on the mineralization process [20], which is known to be an estrogen induced effect. The murine KS483 cell line is a mesenchymal precursor cell line, which differentiates into mature mineralizing osteoblasts during a three-week culture period, when cultured under osteogenesis inducing conditions. This differentiation process can be divided in a proliferation, matrix formation, matrix maturation and finally a mineralization phase, according to the model of Stein and Lian [44], [45]. Thus, the defining characteristic of the mature osteoblast is its ability to produce a mineralized bone matrix. Moreover, KS483 cell model is among few osteoblastic culture systems that can produce discrete, three-dimensionally organized mineralized matrices which are recognizably bone like. These bone nodules consist of woven bone matrix covered by cuboidal osteoblastic cells and containing osteocyte-like cells embedded in the matrix. Characterization of mineralized bone nodules has demonstrated that the processes of nodule formation, matrix deposition and subsequent mineralization follow a well ordered, temporally defined pattern which appears analogous to bone formation and mineralization *in vivo.* Low concentrations of SA or 10H2DA significantly induced mineralization, which was suppressed by the addition of ICI182780, indicating an ER-mediated effect. As expected, the presence of E_2_ significantly stimulated the mineralization of osteoblasts [20]. Our results imply that 10H2DA and SA may be the RJ components that stimulate osteoblasts. None of the FAs stimulated or inhibited cell viability/proliferation of endometrial cancer (Ishikawa) or breast cancer (MFC-7) cells (Fig. S5). The antiestrogenic effect of FAs in breast cancer cells, their favorable effect on osteoblasts and the lack of effect on endometrial cell viability suggest that FAs may be potential natural SERMs.

**Figure 6:**
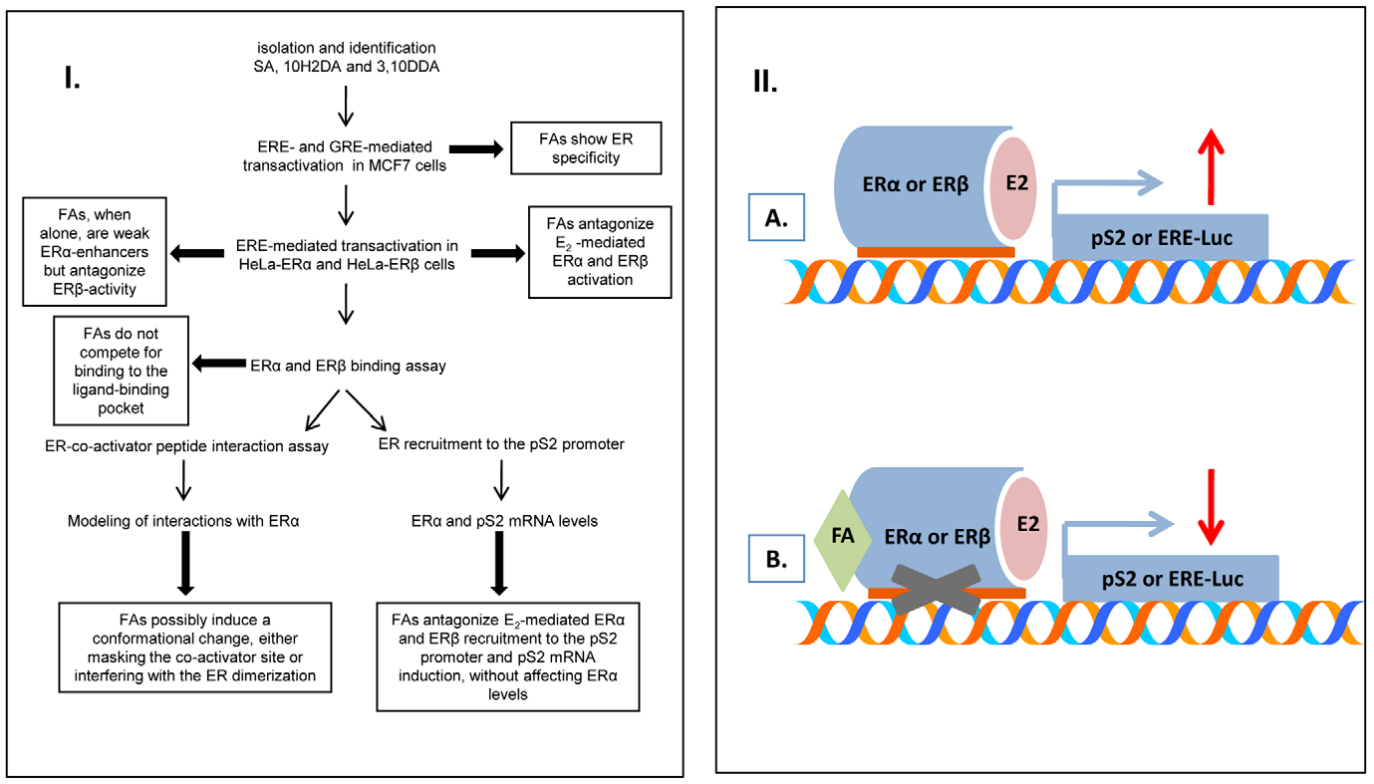
Flow chart of assays and summary of findings. Conclusions are highlighted in lined text boxes (I). Possible molecular mechanism for how FAs modulate E2 signaling through ERs (II). A. Classical E_2_ regulation of gene transcription through recruitment of ERα or ERβ to the promoter region B. In the presence of E_2_, FAs seem to block the effect of E_2_ on ERα and ERβ recruitment to DNA and gene expression (pS2 and ERE-Luc). FAs could bind to a distinct region away from ligand binding pocket either to the co-activator binding pocket or to the dimerization region. This is consistent with the lack of competition by FAs for E_2_ binding to the ligand binding pocket and with the interference of FAs with E_2_ induced binding of a co-activator peptide.

RJ is used extensively in commercial nutritional supplements, medical products, and cosmetics in many countries, while SA, one of its major components, is widely employed in medical practice, e.g. parenteral nutrition, orthopedic applications, drug delivery systems, vaccine development [46], [47], [48], [49], [50]. This honey bee-excreted biological fluid possesses estrogen-like activity, yet the compounds mediating its estrogenic effects are largely unknown. The present report investigated the effects of RJ-derived FAs, namely 10-hydroxy-2-decenoic, 3,10-dihydroxydecanoic and sebacic acid, on estrogen signaling (Fig.6.I.) and suggests that these RJ-derived medium chain fatty acids, structurally entirely different from E_2_, mediate estrogen signaling, at least in part, by modulating the recruitment of ERα, ERβ and co activators to target genes (Fig.6.II.).

## Supporting Information

Figure S1 **Effects of FAs on ERα mRNA and nuclear ERα protein levels in the presence of ERα or ERα and ERβ together.** A–B. MCF-7 tet-off Flag-ERβ cells were treated for 24 hrs with E_2_ (10^−8^ M) or FAs (10H2DA, 3,10DDA, SA) (10^−10^–10^−5^ M). Results are expressed as induction compared to vehicle and normalized to 36B4 mRNA levels. Mean values ± SD are shown from three independent experiments. C–D. MCF-7 tet-off Flag-ERβ cells were treated with vehicle or FAs (10H2DA, 3,10DDA, SA) (10^−5^–10^−6^ M). Cells were harvested 24 hrs later, nuclear extract prepared and ERα detected by Western blotting. β-Actin was used as loading control.
(TIF)

Figure S2 **Effects of FAs on ERE mediated transactivation in MCF-7 cells.** MCF-7 cells were transfected under conditions as shown in Table 2 and treated with FAs (10H2DA, 3,10DDA, SA) (10^−10^–10^−5^ M) alone. Results are normalized to renilla activity and expressed as percentage of luciferase activity in E_2_ incubated samples. Results represent the mean ± SD of 3 independent experiments.
(TIF)

Figure S3 **Effect of DEX, FAs on luciferase activity in MCF-7 cells transfected with a GRE-driven promoter.** MCF-7 cells were transfected under conditions as shown in Table 2 and treated with FAs (10H2DA, 3,10DDA, SA) (10^−10^–10^−5^ M) alone or with the presence of DEX (10^−9^ M). Results of luciferase activity are expressed as percentage of vehicle and normalized to β-galactosidase activity. Columns and bars represent mean value ± SD of the results of three independent experiments.
(TIF)

Figure S4 **Effects of FAs on ERE mediated transactivation in HeLa cells transfected with ERα or ERβ.** HeLa cells were transfected under conditions as shown in Table 2 and treated with E_2_ (10^−9^ M), ICI182780 (10^−8^ M), 4OH-TMX (10^−8^ M) or FAs (10H2DA, 3,10DDA, SA) (10^−10^–10^−5^ M). Co-incubation of ICI 182780 (10^−8^ M) with E_2_ (10^−9^ M) was also done. Results are expressed as percentage of vehicle and normalized to the β-galactosidase activity. Mean values ± SD are shown from the results of three independent experiments. All FAs induced significantly the ERα-mediated Luc activity (significance ranging from p<0.05 to p<0.001), whereas they diminished ERβ-mediated Luc activity (significance ranging from p<0.01 to p<0.001).
(TIF)

Figure S5 **Effect of FAs on cell viability.** MCF-7 (A) and Ishikawa (B) cells were incubated at a concentration range (0.16–400 µΜ) for 48 hrs. Cell viability was determined by the MTT assay. Each point of the dose response curve is the average of four experiments. SD was less than 4% of the average value.
(TIF)
