# Supplementary figures and images for: Fatty Acids Derived from Royal Jelly Are Modulators of Estrogen Receptor Functions
Source: PLoS One. 2010 Dec 22;5(12):e15594. doi: 10.1371/journal.pone.0015594 (PMC3008742; doi:10.1371/journal.pone.0015594)

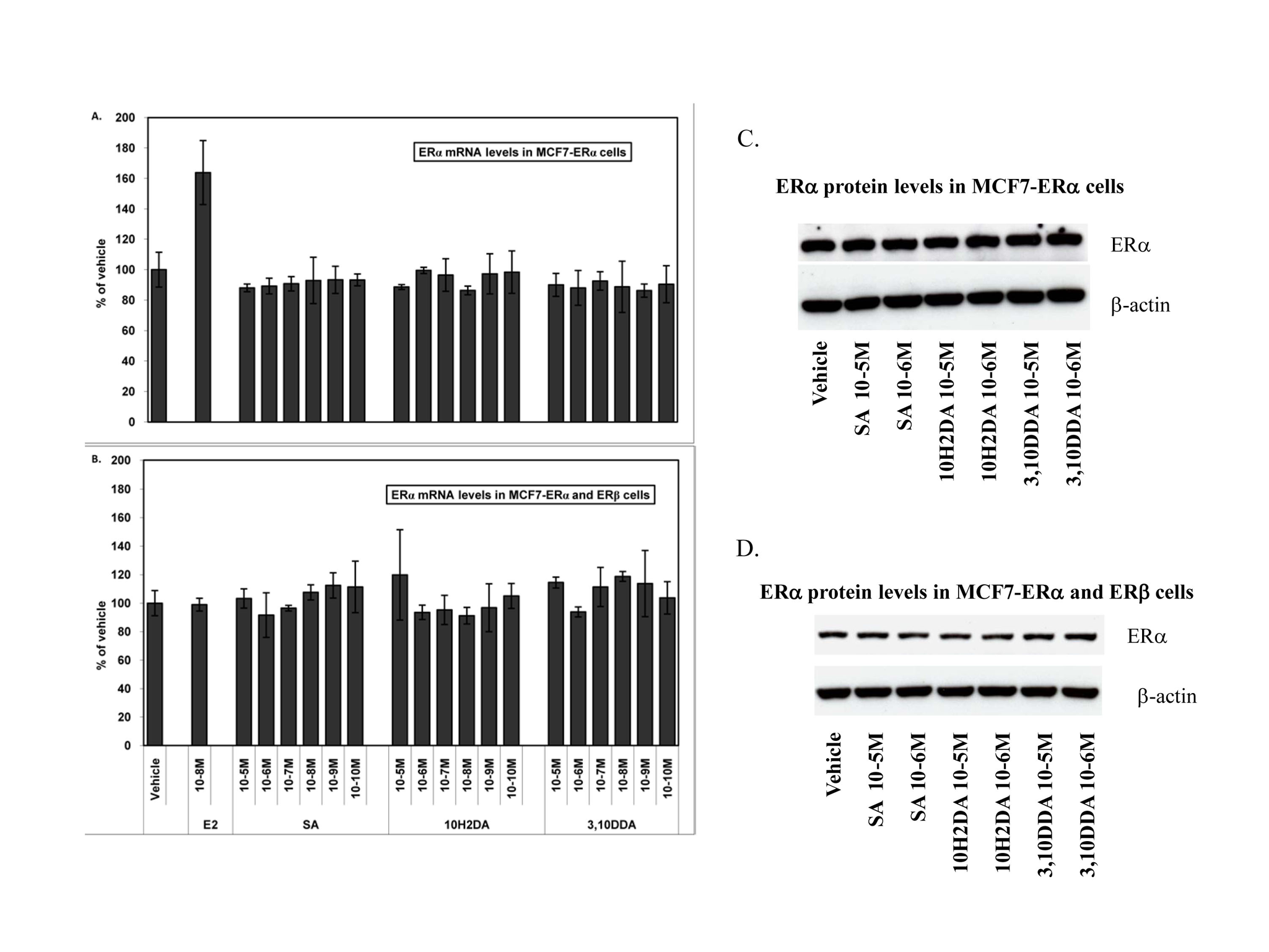

Supplement: Figure S1 — Effects of FAs on ERα mRNA and nuclear ERα protein levels in the presence of ERα or ERα and ERβ together. A–B. MCF-7 tet-off Flag-ERβ cells were treated for 24 hrs with E2 (10−8 M) or FAs (10H2DA, 3,10DDA, SA) (10−10–10−5 M). Results are expressed as induction compared to vehicle and normalized to 36B4 mRNA levels. Mean values ± SD are shown from three independent experiments. C–D. MCF-7 tet-off Flag-ERβ cells were treated with vehicle or FAs (10H2DA, 3,10DDA, SA) (10−5–10−6 M). Cells were harvested 24 hrs later, nuclear extract prepared and ERα detected by Western blotting. β-Actin was used as loading control. (TIF) [file pone.0015594.s001.tif]

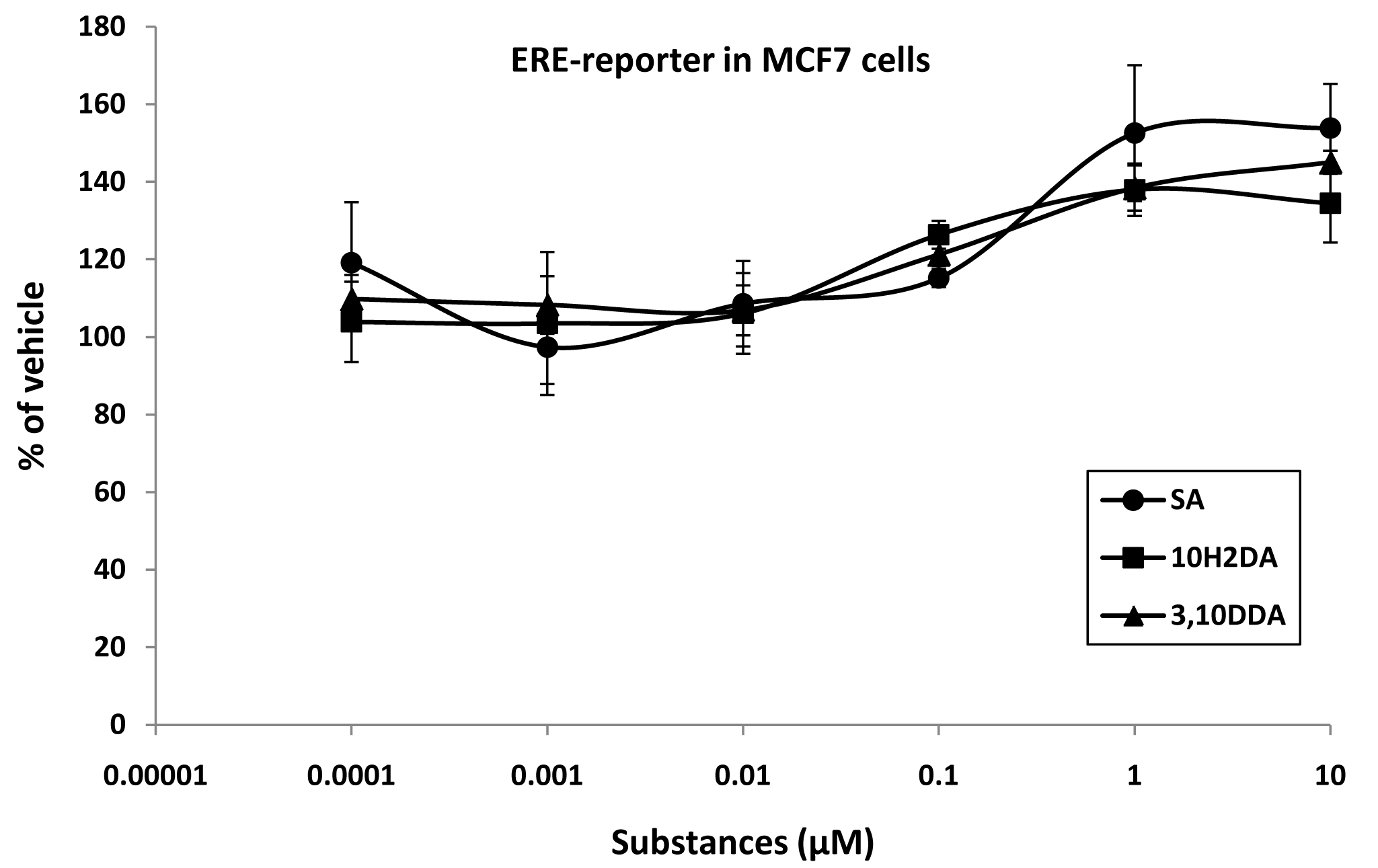

Supplement: Figure S2 — Effects of FAs on ERE mediated transactivation in MCF-7 cells. MCF-7 cells were transfected under conditions as shown in Table 2 and treated with FAs (10H2DA, 3,10DDA, SA) (10−10–10−5 M) alone. Results are normalized to renilla activity and expressed as percentage of luciferase activity in E2 incubated samples. Results represent the mean ± SD of 3 independent experiments. (TIF) [file pone.0015594.s002.tif]

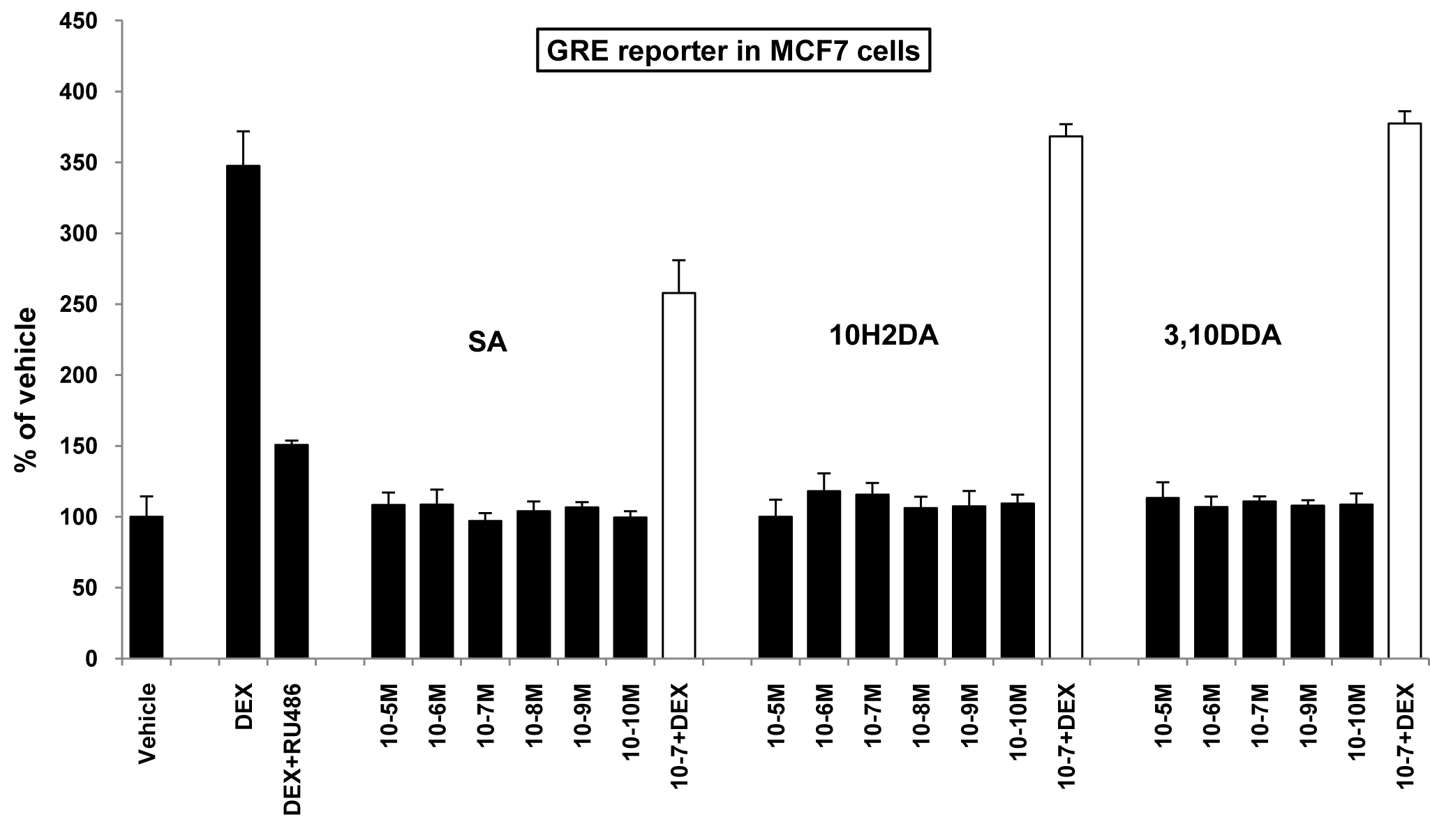

Supplement: Figure S3 — Effect of DEX, FAs on luciferase activity in MCF-7 cells transfected with a GRE-driven promoter. MCF-7 cells were transfected under conditions as shown in Table 2 and treated with FAs (10H2DA, 3,10DDA, SA) (10−10–10−5 M) alone or with the presence of DEX (10−9 M). Results of luciferase activity are expressed as percentage of vehicle and normalized to β-galactosidase activity. Columns and bars represent mean value ± SD of the results of three independent experiments. (TIF) [file pone.0015594.s003.tif]

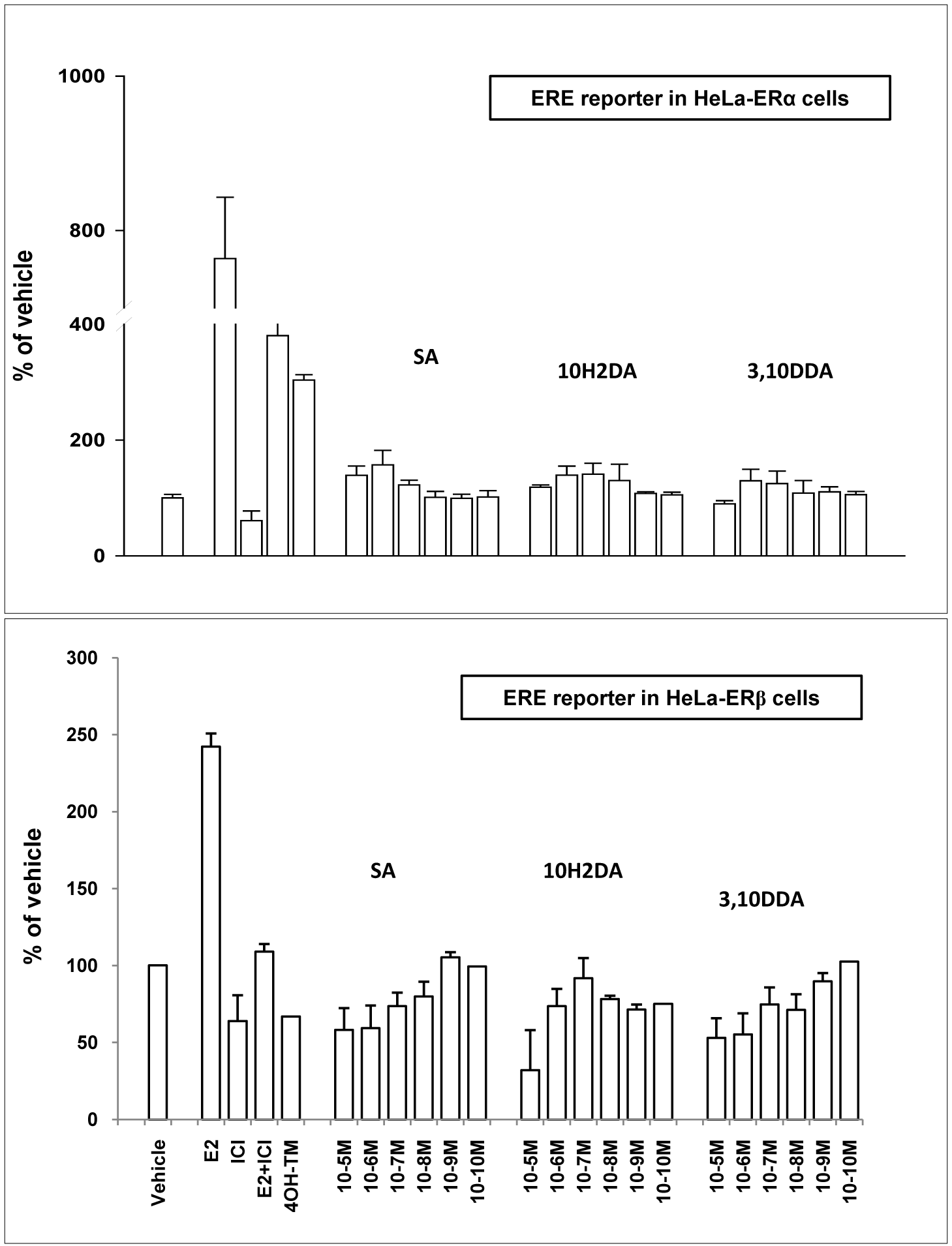

Supplement: Figure S4 — Effects of FAs on ERE mediated transactivation in HeLa cells transfected with ERα or ERβ. HeLa cells were transfected under conditions as shown in Table 2 and treated with E2 (10−9 M), ICI182780 (10−8 M), 4OH-TMX (10−8 M) or FAs (10H2DA, 3,10DDA, SA) (10−10–10−5 M). Co-incubation of ICI 182780 (10−8 M) with E2 (10−9 M) was also done. Results are expressed as percentage of vehicle and normalized to the β-galactosidase activity. Mean values ± SD are shown from the results of three independent experiments. All FAs induced significantly the ERα-mediated Luc activity (significance ranging from p<0.05 to p<0.001), whereas they diminished ERβ-mediated Luc activity (significance ranging from p<0.01 to p<0.001). (TIF) [file pone.0015594.s004.tif]

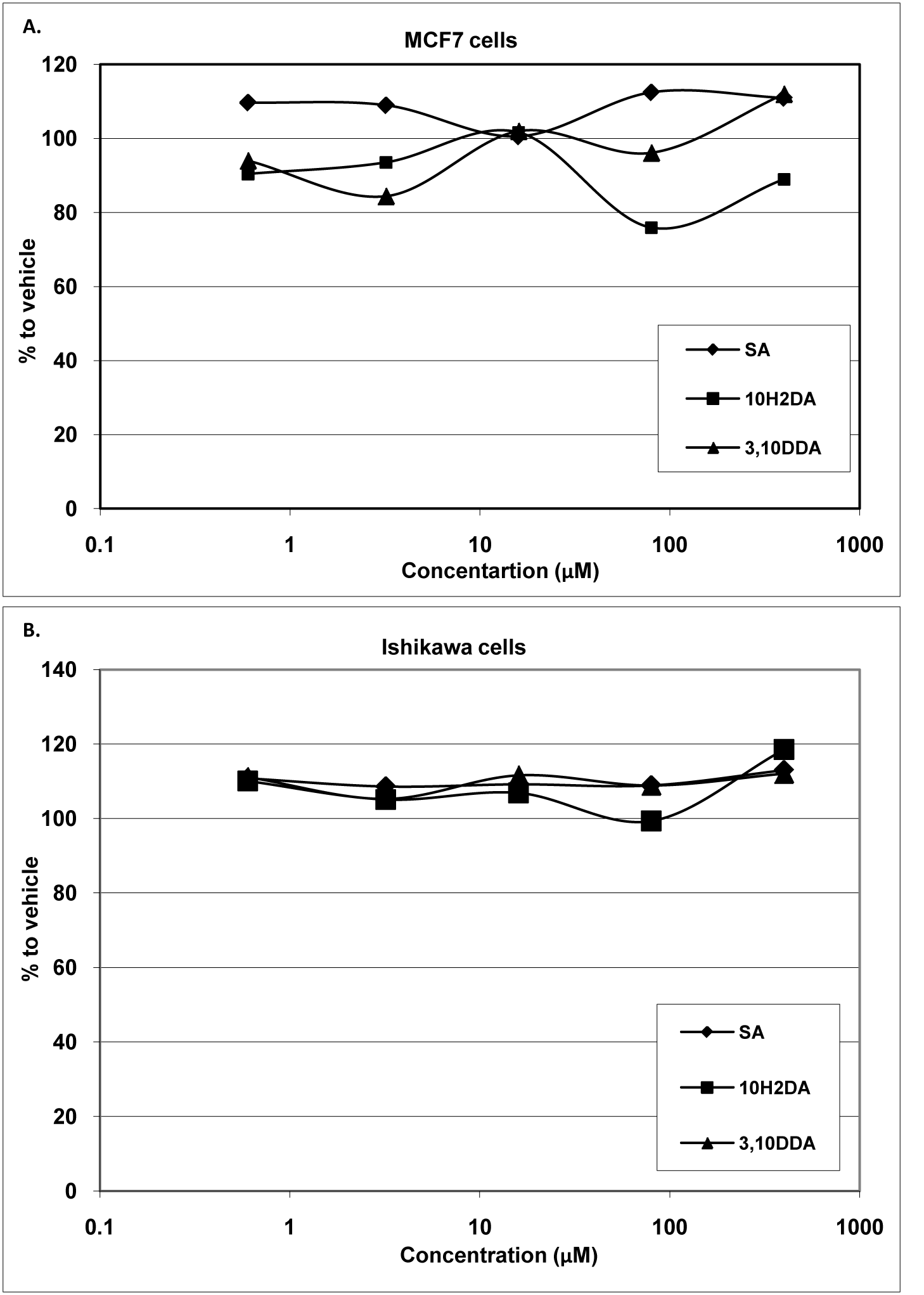

Supplement: Figure S5 — Effect of FAs on cell viability. MCF-7 (A) and Ishikawa (B) cells were incubated at a concentration range (0.16–400 µΜ) for 48 hrs. Cell viability was determined by the MTT assay. Each point of the dose response curve is the average of four experiments. SD was less than 4% of the average value. (TIF) [file pone.0015594.s005.tif]
